# Impact of asthma on women and men: Comparison with the general population using the EQ-5D-5L questionnaire

**DOI:** 10.1371/journal.pone.0202624

**Published:** 2018-08-23

**Authors:** Gimena Hernandez, Alexandra L. Dima, Àngels Pont, Olatz Garin, Marc Martí-Pastor, Jordi Alonso, Eric Van Ganse, Laurent Laforest, Marijn de Bruin, Karina Mayoral, Montse Ferrer, the ASTRO-LAB group

**Affiliations:** 1 Health Services Research Group, IMIM (Hospital del Mar Medical Research Institute), Barcelona, Spain; 2 Dept of Paediatrics, Obstetrics and Gynaecology and Preventive Medicine, Universitat Autònoma de Barcelona, Bellaterra, Barcelona, Spain; 3 CIBER en Epidemiología y Salud Pública, CIBERESP, Madrid, Spain; 4 Amsterdam School of Communication Research ASCoR, University of Amsterdam, Amsterdam, The Netherlands; 5 Pompeu Fabra University (UPF), Barcelona, Spain; 6 PharmacoEpidemiology Lyon (PEL), HESPER Unit, Claude-Bernard University-Lyon1, Lyon, France; 7 Respiratory Medicine, Croix-Rousse Hospital, CHU-Lyon, Lyon, France; 8 Institute of Applied Health Sciences, University of Aberdeen, Aberdeen, Scotland, United Kingdom; National and Kapodistrian University of Athens, GREECE

## Abstract

**Background:**

The aim was to evaluate the impact of asthma on patients’ Health-Related Quality of Life (HRQoL) by comparing asthmatic women and men with reference norms, to examine the factors which contributed to an impaired HRQoL, and to identify groups at higher risk.

**Methods:**

Cross-sectional evaluation of 222 primary care patients with persistent asthma (18–40 years old). HRQoL impact was estimated with the EuroQol-5 Dimensions (EQ-5D), which allows calculating Quality-Adjusted Life-Years (QALYs) by applying society preferences. Participants self-completed the EQ-5D questionnaire online. Telephonic interviews collected information on medication and adherence, and administered the Asthma Control Questionnaire. Severity markers included asthma-related comorbidity, previous oral corticosteroids course prescription, and inhaled corticosteroids daily dose. After bivariate analyses, multiple linear regression models were constructed to examine the relations between HRQoL asthma impact and socio-demographic and clinical variables, using as dependent variable the deviation from general population-based EQ-5D reference norms.

**Results:**

Deviation from the EQ-5D index norms was moderate in most age/gender groups (-0.1, which corresponds to 0.6 standard deviations), while it was large in women aged 18–24 years (-0.18, corresponding to 1.1 standard deviations). In regression models, a poor asthma control was the only factor independently associated to HRQoL impact in both women and men: β -0.18 (p<0.001) and -0.15 (p = 0.01) respectively. Translating these β coefficients to QALYs, they are interpretable as 66 fewer days of full health per year in women with uncontrolled asthma and 55 for men, compared with those with controlled asthma.

**Conclusion:**

Persistent asthma has a moderately negative HRQoL impact on patients of both genders, and the youngest women have been identified as a high risk group which merits further research. We identified asthma control as the major contributor to impaired HRQoL in patients, regardless of their gender, suggesting that asthma HRQoL impact could be alleviated by achieving a good control of symptoms.

## Introduction

International guidelines for asthma have emphasized the need to include patients´ Health-Related Quality of Life (HRQoL) [1] improvement in treatment goals. Studies on clinical samples have reported worse HRQoL in women with asthma, compared with men [2–4]. Significant gender differences in lifespan among people with asthma have also been documented, and asthma-related hospitalizations were found to be most prevalent among middle-aged women [5]. Could these differences imply gender inequalities in HRQoL asthma impact? Clinical studies offer limited information on this topic because they lack a comparison with the general population, where women were also found to have worse HRQoL than men [6–8]. Therefore, to answer this question, we need to know how far the HRQoL of asthma patients is from the general population, by comparing them with controls or reference norms.

The instruments used to assess HRQoL can be roughly divided into disease-specific and generic ones [9]. While the former are very useful, they do not usually allow the evaluation of asthma impact in comparison with that of other diseases or with the general population. Reference norms have been mainly developed to interpret generic HRQoL questionnaires, permitting comparisons of a disease-specific sample with counterparts from the general population. This approach has been successfully applied in diseases such as fibromyalgia and rheumatoid arthritis [10], thalassemia [11], epilepsy [12], and type 2 diabetes [13]. To our knowledge, there are no studies that have assessed asthma impact on HRQoL using reference norms.

There are some studies based on National Health Surveys, but they usually evaluate individuals who self-reported having asthma and, thus, the lack of a reliable diagnosis might have led to under- or over-estimating asthma impact on their HRQoL. The 2000 Behavioral Risk Factor Surveillance System included 12,270 individuals with self-reported asthma who perceived worse HRQoL than those who had never had asthma [14], administering four HRQoL questions but without any standardized instrument. The 2008 European National Health and Wellness Survey, with the 12-item Short-Form Health Survey (SF-12) [15], showed worse results among the 3,619 individuals with self-reported asthma than among general population.

The EuroQol 5 Dimensions (EQ-5D), one of the most widely employed generic tools due to its low respondent burden and good psychometric properties [16–19], has reference norms for 24 countries [20]. Furthermore, the EQ-5D allows the calculation of Quality-Adjusted Life-Years (QALYs) when society preferences are applied [21]. The aim of this study was to evaluate the impact of asthma on patients’ HRQoL by comparing asthmatic women and men with EQ-5D reference norms, to examine the factors which contributed to an impaired HRQoL, and to identify specific groups at higher risk.

We hypothesised that worse HRQoL in women with asthma compared with men [2–4] does not imply gender inequalities in asthma impact, because their worse HRQoL is mainly explained by gender differences external to asthma, such as other chronic conditions, disease-related behaviours, or socio-economic background. In this sense, we expected that when asthma impact on HRQoL is defined as the deviation from general population-based reference norms, differences between women and men with asthma would disappear. According to the available evidence [22–27], we also hypothesised that the main factors related to the HRQoL of asthmatic patients were age, socio-economic characteristics (education, work status,…), smoking status, asthma control, controller and reliever medication, adherence to inhalers, comorbidities, and severity.

## Materials and methods

### Setting and study population

We analysed baseline data from French adult patients (18–40 years old) with persistent asthma who completed the EQ-5D questionnaire with 5 levels (EQ-5D-5L) in the ASTRO-LAB project, approved by the Ethics and Regulatory Boards, and conducted in accordance with the Declaration of the World Medical Association. CCTIRS (Comité consultatif sur le traitement de l'information en matière de recherche dans le domaine dela santé) approval was obtained on November 21st, 2012 (Dossier N°12702); and CNIL (Commission Nationale d’Informatique et Liberté) the authorization was obtained in May 17th, 2013 (DR-2013-264). Written informed consent was obtained from all French participants prior to inclusion.

The ASTRO-LAB project was designed as a prospective longitudinal study to evaluate the safety of long-acting beta-agonists (LABAs). Patients were enrolled in primary care in France and United Kingdom by their general practitioner, based on 12-month prescription data. Inclusion criteria were: subjects aged 6–40 years with persistent asthma defined as more than 6 months of prescribed inhaled corticosteroids and/or LABAs during 12 months before inclusion. Exclusion criteria were: chronic oral corticosteroid use (≥15 consecutive days during 3 months before inclusion), history of omalizumab therapy, and/or any other concomitant chronic respiratory disease (chronic obstructive pulmonary disease, cystic fibrosis, pulmonary fibrosis, bronchiectasis or tuberculosis). In addition to clinical records, the main information sources of ASTROLAB were: computer-assisted telephone interviews (CATIs), mobile text messages, and online surveys.

Trained interviewers administered CATIs to patients after inclusion, and then every four months during a follow-up of 24 months at maximum. CATIs assessed asthma medications prescribed, their patient-reported use, control of symptoms, and the occurrence of asthma exacerbations during the previous 4 months. Patients received monthly mobile text messages inquiring whether they had experienced a new asthma exacerbation since the last study contact. Positive responses motivated an extra CATI to characterize the exacerbation. Patients were also requested to complete an online survey at inclusion and at 12-month intervals on socio-demographic characteristics, determinants of medication adherence, triggers, exacerbations management, quality of inhaler technique, and EQ-5D questionnaire. The complete ASTRO-LAB protocol is available in a previous publication [28].

### Measurement instruments

General practitioners completed an online survey at patient recruitment with information on age, commonly asthma-associated conditions, and medications prescribed during the 12 months before inclusion. The history of allergic rhinitis, nasal polyps, infectious sinusitis, anxiety/depression, and gastro-esophageal reflux was registered and transformed into a count variable as a summary indicator of asthma-related comorbidity, as well as the number of prescribed oral corticosteroids courses 12 months before inclusion. These two variables, together with the daily dose of inhaled corticosteroids, were used as severity markers.

#### Patient-reported data collected by computer-assisted telephonic interviews (CATI)

We used data from the first (baseline) CATI, which included the Asthma Control Questionnaire-symptoms only (ACQ), and questions on type and adherence to daily controller medication, reliever medication, and the daily dose of inhaled corticosteroids prescribed at the time of inclusion (beclomethasone equivalent). The latter was categorized following clinical guidelines [29] into high (>1,000μg), medium (500 to 1,000 μg), and low (≤500 μg).

The ACQ–symptoms only [30] assesses the frequency of five asthma symptoms during the previous week through Likert scales with 7 response options. The overall score, calculated as the mean of item responses, ranges from 0 to 6. A score <0.75 is defined as well-controlled asthma; 0.75–1.5 as intermediate asthma control; and >1.5 as not well-controlled asthma [31].

Adherence to daily controller medication was measured with the Medication Intake Survey-Asthma (MIS-A) [32], a count-based recall measure of medication implementation. MIS-A 1-week adherence was estimated by the proportion of prescribed medication that the patient had used the previous week. It was categorized into complete (100%), intermediate, and low (≤50%) adherence.

Reliever medication in the past month was measured with the following question: ‘How often have you usually taken your (brand name) in the past 4 weeks? Every day; almost every day; once or twice every week; or less than once a week’. Responses were dichotomized according to the cut-off point of more than twice per week [29].

#### EQ-5D-5L and socio-demographic variables

At study enrollment, patients were invited to self-complete an online survey, which included among others the EQ-5D-5L to measure HRQoL, smoking status, and socio-demographic data on highest education and work status.

The EQ-5D-5L is a brief, multi-attribute, generic, health status measure composed of a descriptive system and a visual-analogue scale (EQ-VAS) asking individuals to rate their own health from 0 to 100 (worst and best imaginable health, respectively). The descriptive system covers five dimensions of health (mobility, self-care, usual activities, pain/discomfort, and anxiety/depression) with five response options in each dimension (no problems, slight problems, moderate problems, severe problems, unable to perform/extreme problems). The EQ-5D-5L therefore defines 3125 distinct health states from all the possible combinations of dimensions and response options (i.e. 5^5^). Each of these combinations was converted into a single health index ranging from 1 (the best health state) to negative values (health states valued as worse than death) where 0 is equal to death. This conversion was performed applying a formula that attaches societal preference values (weights) to each response. The index was calculated with the crosswalk 3L-5L French value set of preferences [33,34].

### Analytic strategy

We calculated the statistical power to estimate the mean of the EQ-5D health index with a 95% confidence interval precision of +/-0.07, which was the Minimal Important Difference (MID) previously established [35]. Given a standard deviation of 0.16, statistical power was 0.80 for the smallest group of our sample (18–25 years old men, n = 19).

Reference norms published by the EuroQol group [20] for France were obtained from a representative sample of non-institutionalized adults [36]. Deviation from reference norms for the EQ-5D-5L index and the EQ-VAS were calculated by subtracting the patients’ mean from the mean of their corresponding age and gender group, and negative values indicate worse health than counterparts from the general population.

All the analyses were carried out separately for women and men. Comparisons among groups were made using chi-squared tests for categorical variables and ANOVA for continuous variables. Multiple linear regression models were constructed to examine the relation of asthma HRQoL impact with socio-demographic and clinical variables, using EQ-5D-5L index and EQ-VAS deviation from reference norms as dependent variables. The covariates were chosen a priori, based on knowledge about determinants of HRQoL in asthma. Analyses were conducted using the statistical package SPSS12, and α was set at 0.05.

## Results

Of the 487 French subjects with asthma aged 18–40 years from the ASTRO-LAB cohort, 245 (50.3%) filled in the baseline online survey; 23 did not complete the EQ-5D-5L questionnaire, hence 222 participants were included in the analysis.

Patients had a mean age of 30.3 years (SD 6.7), 61.3% were women, 72% were currently employed, and 63% were non-smokers (Table 1). The means of the EQ-5D-5L index and EQ-VAS were 0.83 and 77.3, respectively, and deviations from reference norms were -0.11 and -4.9. Asthma control was evenly distributed among the three categories. Most patients were prescribed ICs/LABA fixed-dose-combinations, and 43% reported complete adherence. Severity markers showed that 58.5% presented one or more asthma-related comorbidities, around 25% used a high inhaled corticosteroids dose, and 30% was prescribed at least one oral corticosteroid course during the previous 12 months. Statistically significant differences between genders were observed for education (p = 0.019), inhaled corticosteroids daily dose prescription (p = 0.005) and the number of oral corticosteroids courses prescribed (p = 0.002), which indicated more severe asthma for women than men. All EQ-5D results showed a worse HRQoL in women.

**Table 1:** Characteristics of study subjects.

	Total (n = 222)	Women (n = 136)	Men (n = 86)	p-value
Age, mean (SD)	30.3 (6.7)	29.7 (6.6)	31.3 (6.7)	0.079
18–24 years	55 (24.8%)	36 (26.5%)	19 (22.1%)	0.124
25–35 years	97 (43.7%)	64 (47.1%)	33 (38.4%)	
35 or more years	70 (31.5%)	36 (26.5%)	34 (39.5%)	
Highest education				
Sixth form or college, Secondary or less	30 (13.8%)	11 (8.2%)	19 (22.6%)	0.019
Bachelor Degree	59 (27.1%)	40 (29.9%)	19 (22.6%)	
Bachelor Degree +2 or +3	98 (45.0%)	61 (45.5%)	37 (44.0%)	
Bachelor Degree +5 or more	31 (14.1%)	22 (16.4%)	9 (10.7%)	
Work status				
Employed at usual job	158 (71.8%)	91 (67.4%)	67 (78.8%)	0.168
Paid sick leave, restricted work, light duty due to disability	9 (4.1%)	7 (5.2%)	2 (2.4%)	
Not working for other reason	53 (24.1%)	37 (27.4%)	16 (18.8%)	
Smoking status				
Non Smoker	137 (62.8%)	88 (66.2%)	49 (57.6%)	*0*.*204*
Smoker	81 (37.2%)	45 (33.8%)	36 (42.4%)	
**Patient-Reported Outcomes**				
EuroQol-5 Dimensions-5 Levels (EQ-5D-5L), mean (SD)				
EQ-5D-5L Index	0.83 (0.17)	0.81 (0.18)	0.86 (0.15)	0.016
EQ-5D-5L index deviation from Reference norms	-0.11 (0.17)	-0.13 (0.19)	-0.07 (0.15)	0.015
EQ- VAS	77.3 (16.5)	76.1 (18.5)	79.2 (12.4)	0.137
EQ-VAS deviation from Reference norms	-4.9 (16.8)	-6.7 (18.8)	-2.0 (12.5)	0.045
Asthma Control Questionnaire (ACQ), mean (SD)	1.1 (1.0)	1.2 (1.0)	1.0 (0.9)	0.076
Well controlled (< 0.75)	67 (37.9%)	36 (33.3%)	31 (44.9%)	0.281
Intermediate (0.75–1.5)	61 (34.5%)	39 (36.1%)	22 (31.9%)	
Not well controlled (> 1.5)	49 (27.7%)	33 (30.6%)	16 (23.2%)	
**Asthma medication**				
Type of controller medication				
Inhaled corticosteroids (ICs)	39 (17.6%)	23 (16.9%)	16 (18.6%)	0.781
Long-acting beta-agonists (LABA) with/out ICs	30 (13.5%)	17 (12.5%)	13 (15.1%)	
ICs/LABA Fixed-dose combination	153 (68.9%)	96 (70.6%)	57 (66.3%)	
Adherence (MIS-A 1-week)				
Low (≤ 50%)	57 (30.3%)	*36 (30*.*3%)*	21 (30.4%)	0.687
Intermediate	50 (26.6%)	34 (28.6%)	16 (23.2%)	
Complete (100%)	81 (43.1%)	*49 (41*.*2%)*	32 (46.4%)	
Reliever medication use				
Never	56 (26.5%)	36 (27.9%)	20 (24.4%)	0.395
Less than once a week	79 (37.4%)	51 (39.5%)	28 (34.1%)	
Once or twice every week	54 (25.6%)	32 (24.8%)	22 (26.8%)	
Almost every day	22 (10.4%)	10 (7.8%)	12 (14.6%)	
**Severity Markers**				
Asthma-related comorbidities				
0	66 (41.5%)	38 (38.4%)	28 (46.7%)	0.443
1	62 (39.0%)	39 (39.4%)	23 (38.3%)	
2 or more	31 (19.5%)	22 (22.2%)	9 (15.0%)	
Inhaled Corticosteroids daily dose^1^, mean (SD)	929.8 (866.2)	1051.2 (960.9)	728.4 (637.2)	0.005
≤ 500 μcg	89 (44.1%)	50 (39.7%)	39 (51.3%)	0.094
500–1000 μcg	65 (32.2%)	40 (31.7%)	25 (32.9%)	
> 1000 μcg	48 (23.8%)	36 (28.6%)	12 (15.8%)	
Oral Corticosteroids courses^2^, mean (SD)	0.4 (0.8)	0.6 (0.9)	0.3 (0.6)	0.004
0 courses	152 (70.4%)	82 (62.6%)	70 (82.4%)	0.002
1 or more courses	64 (29.6%)	49 (37.4%)	15 (17.6%)	

1 Inhaled corticosteroids prescribed at the time of inclusion (beclomethasone equivalent)

2 Oral corticosteroids courses prescribed during the 12 months before inclusion

**Fig 1:**
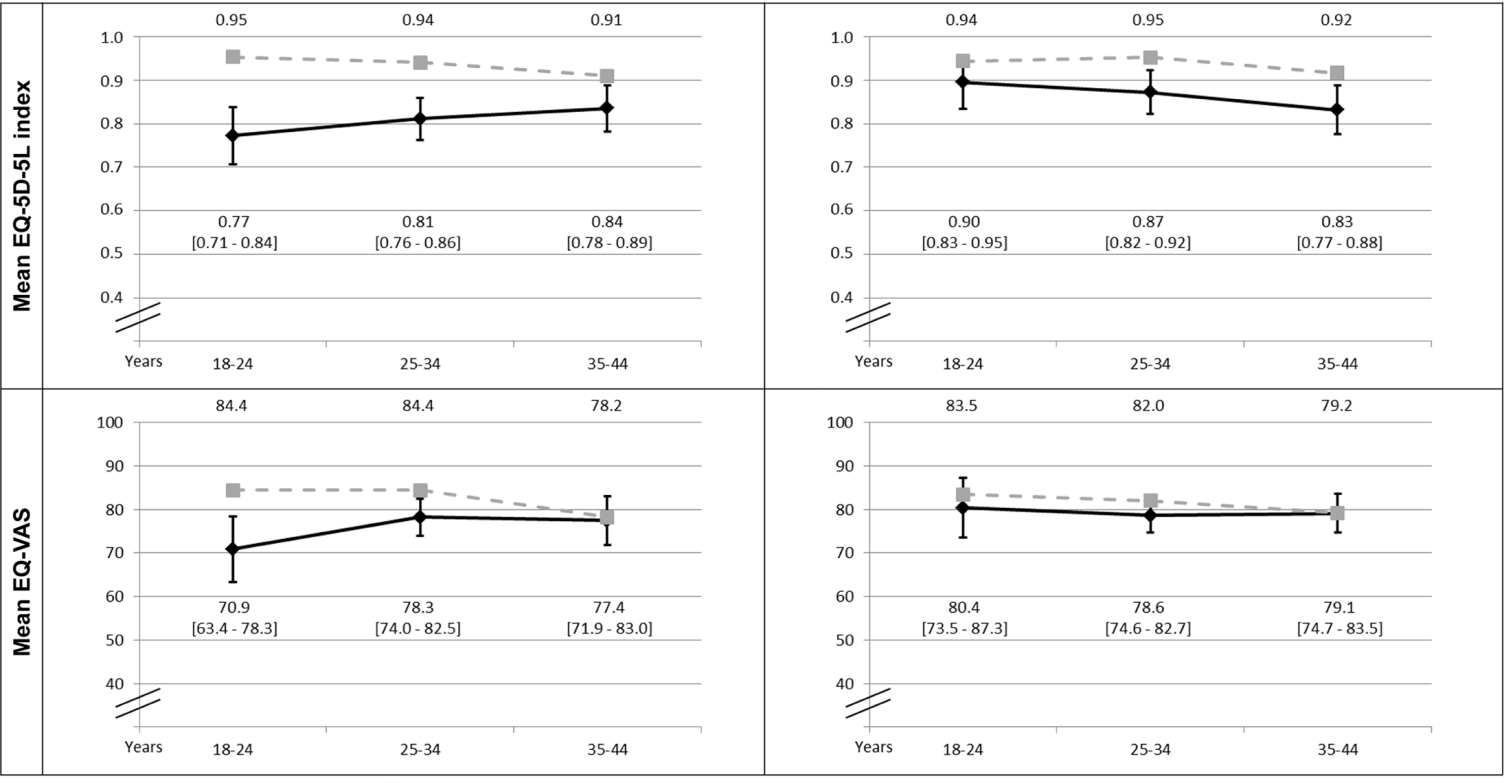
EQ-5D index and EQ-VAS: comparison between patients with asthma and French general population-based reference norms. Mean and 95% Confidence Interval (95%CI) of EQ-5D index and EQ-VAS in patients with asthma stratified by age and gender (in black). Grey dotted line represents the mean in French general population-based reference norms [20].

French reference population norms and EQ-5D results in women and men with asthma are shown in (Fig 1A and 1B respectively). Mean EQ-5D index in asthmatic women (Fig 1A) was 0.77 (95%CI 0.71–0.84) for those aged 18–24, 0.81 (95%CI 0.76–0.85) for those aged 25–34, and 0.83 (95%CI 0.78–0.88) for those aged 35–40. All these means were significantly different from norms, as the 95% CI didn´t include the mean of the reference norm in any age group. For example, the mean value for women aged 18–24 in the general population was 0.95 [20], which was clearly outside of the 95% CI found in asthmatic women of this age (mean = 0.77, 95%CI 0.71–0.84). The differences between reference norms and the results obtained among women with asthma were markedly greater in the youngest, and they diminished with age (Fig 1A): -0.18, -0.13, and -0.075, respectively. In contrast to the women’s pattern, differences on EQ-5D index between men with asthma and reference norms increased slightly with age (-0.05, -0.08, and -0.085, respectively), and were statistically significant for the two oldest groups (Fig 1B). EQ-VAS showed that younger women (18–24 years) perceived significantly worse health than their counterparts, while men with asthma were very close to reference norms.

**Fig 2:**
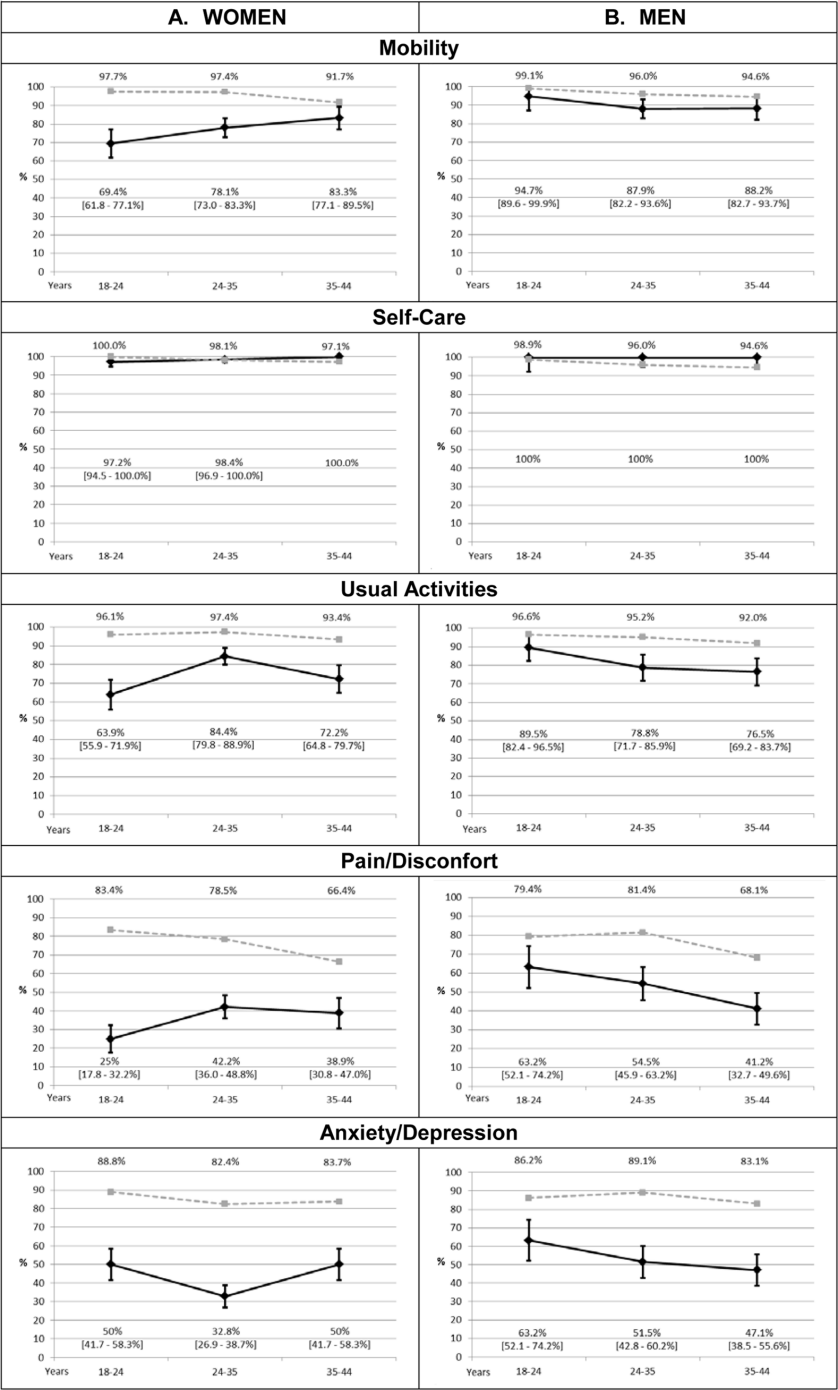
EQ-5D dimensions: Comparison between patients with asthma and French general population-based reference norms. Percentage and 95% Confidence Interval (95%CI) of problems in each EQ-5D dimension reported by patients with asthma (in black). Grey dotted line represents the percentage in French general population-based reference norms [20].

Fig 2 shows that the proportion of women and men with asthma reporting problems is higher than reference norms in usual activities, pain/discomfort, and anxiety/depresion. The youngest women also reported more problems in mobility.

Deviations from reference norms for EQ-5D-5L index and EQ-VAS in socio-demographics and clinical groups are presented in Table 2. Negative values indicate that all asthmatic groups presented worse health than their counterparts from the general population. These negative values were always larger in women than men. Among women the biggest deviation from reference norms was found in those using reliever medication almost or every day (mean = -0.31), followed by those with not well-controlled asthma (mean = -0.28), those with 2 or more asthma-related comorbidities (mean = -0.22), and those with inhaled corticosteroids daily dose >1000 μcg (mean = -0.21). Among men, EQ-5D-5L index deviation from norms only showed statistically significant differences regarding asthma control and reliever medication use. The EQ-VAS deviations from reference norms were significantly associated with age, asthma control, and severity markers in women, but only with asthma control in men.

**Table 2:** Mean (SD) of deviations from reference norms: EQ-5D-5L index and EQ-VAS.

	EQ-5D-5L deviation from reference norm		EQ-VAS deviation from reference norm	
	Women	Men	Women	Men
Age				
18–24 years	-0,18 (0,20)	-0,05 (0,13)	-13,54 (22,11)	-3,13 (14,30)
25–35 years	-0,13 (0,19)	-0,08 (0,14)	-6,12 (17,02)	-3,36 (11,33)
35 or more years	-0,08 (0,16)	-0,08 (0,16)	-0,76 (16,43)	-0,11 (12,71)
p-value	0.055	0.690	0.014	0.523
Highest education				
Not Universitary	-0,14 (0,19)	-0,09 (0,16)	-6,09 (21,37)	-1,75 (14,46)
Universitary	-0,12 (0,19)	-0,06 (0,14)	-7,20 (17,39)	-2,22 (11,14)
p-value	0.589	0.366	0.744	0.866
Smoking status				
Non-Smoker	-0,13 (0,18)	-0,05 (0,14)	-7,15 (19,33)	-1,77 (12,96)
Smoker	-0,12 (0,17)	-0,10 (0,15)	-4,77 (15,78)	-2,38 (12,25)
p-value	0.728	0.128	0.477	0.825
**Patient-Reported Outcomes**				
Asthma control Questionnaire (ACQ)				
Well controlled (< 0.75)	-0,04 (0,13)	-0,01 (0,07)	-2,53 (15,02)	-0,94 (10,98)
Intermediate (0.75–1.5)	-0,14 (0,15)	-0,10 (0,15)	-4,15 (11,26)	0,02 (14,33)
Not well controlled (> 1.5)	-0,28 (0,22)	-0,18 (0,17)	-16,23 (21,73)	-9,85 (13,38)
p-value	< 0.001	< 0.001	0.001	0.041
**Asthma medication**				
Type of controller medication				
Inhaled Corticosteroids (ICs)	-0,11 (0,15)	-0,05 (0,18)	-4,18 (16,89)	-3,81 (13,42)
Long-acting beta-agonist (LABA) with/out ICs	-0,12 (0,16)	-0,09 (0,16)	-7,76 (22,90)	1,94 (13,18)
ICs/LABA fixed combination	-0,14 (0,20)	-0,08 (0,14)	-7,06 (18,63)	-2,43 (12,16)
p-value	0.853	0.753	0.781	0.435
Adherence (MIS-A 1-week)				
Low (≤50%)	-0,14 (0,20)	-0,07 (0,16)	-6,55 (16,13)	-3,58 (15,95)
Intermediate	-0,15 (0,17)	-0,13 (0,17)	-3,84 (13,61)	-4,63 (10,70)
Complete (100%)	-0,09 (0,15)	-0,07 (0,12)	-7,91 (22,43)	-1,36 (13,16)
p-value	0.274	0.345	0.611	0.700
Reliever medication use				
Twice a week or less	-0,12 (0,17)	-0,06 (0,13)	-6,54 (16,10)	-1,51 (12,08)
More than twice a week	-0,31 (0,30)	-0,17 (0,18)	-15,56 (25,68)	-8,36 (16,68)
p-value	0.002	0.010	0.155	0.065
**Severity markers**				
Asthma-related comorbidities				
0	-0,09 (0,16)	-0,05 (0,12)	-4,57 (17,54)	-0,80 (9,97)
1	-0,11 (0,15)	-0,06 (0,14)	-3,46 (12,90)	-3,25 (14,70)
2 or more	-0,22 (0,22)	-0,11 (0,13)	-11,73 (19,57)	-4,76 (11,27)
p-value	0.013	0.460	0.149	0.631
Inhaled Corticosteroids daily dose^1^				
≤ 500 μcg	-0,11 (0,18)	-0,05 (0,12)	-6,60 (17,45)	-0,17 (10,69)
500–1000 μcg	-0,09 (0,18)	-0,10 (0,17)	-1,44 (16,96)	-2,60 (13,67)
> 1000 μcg	-0,21 (0,20)	-0,11 (0,16)	-13,89 (21,74)	-5,75 (13,53)
p-value	0.011	0.338	0.016	0.362
Oral Corticosteroids courses^2^				
0	-0,11 (0,17)	-0,07 (0,14)	-3,81 (14,18)	-1,95 (12,96)
1 or more	-0,16 (0,21)	-0,10 (0,18)	-10,68 (23,47)	-2,91 (10,89)
p-value	0.177	0.454	0.039	0.789

1 Inhaled corticosteroids prescribed at the time of inclusion (beclomethasone equivalent)

2 Oral corticosteroids courses prescribed during the 12 months before inclusion

Table 3 presents linear regression models with deviations from reference norms for EQ-5D index and EQ-VAS as dependent variables. Among women, a significant relationship with asthma control (β -0.18 for not well-controlled, p<0.001) and adherence (β -0.10 for low adherence, p = 0.03) was found. In men, only those with not well-controlled asthma presented higher deviation from norms (indicating worse health), compared with well-controlled asthma (ß = -0.15, p = 0.01). Regression models with EQ-VAS only showed a significantly worse perceived health in women with uncontrolled asthma (p = 0.028), and with inhaled corticosteroids daily dose ≤ 500 μg (p = 0.012).

**Table 3:** Regression models of EQ-5D-5L and EQ-VAS deviation from norms regarding gender.

	EQ-5D Deviation				VAS Deviation			
	Women		Men		Women		Men	
	β (95% CI)	P	β (95% CI)	P	β (95% CI)	p	β (95% CI)	P
(Constant)	-0.05 (-0.21, 0.10)	0.490	0.04 (-0.20, 0.28)	0.760	-5.61 (-19.84, 8.63)	0.434	-13.96 (-37.50, 9.58)	0.237
*Age*								
*18–24 years*	*Reference*		*Reference*		*Reference*		*Reference*	
25–35 years	0.03 (-0.05, 0.11)	0.466	-0.04 (-0.19, 0.12)	0.624	3.74 (-4.01, 11.49)	0.339	7.15 (-8.19, 22.49)	0.351
35 or more years	0.03 (-0.07, 0.14)	0.547	-0.05 (-0.19, 0.10)	0.517	7.24 (-2.67, 17.16)	0.149	10.23 (-3.72, 24.17)	0.146
*Highest education*								
Not Universitary	*Reference*		*Reference*		*Reference*		*Reference*	
Universitary	0.00 (-0.07, 0.08)	0.926	0.02 (-0.07, 0.10)	0.653	-4.87 (-12.15, 2.41)	0.186	1.70 (-6.67, 10.08)	0.683
*Smoking status*								
Non smoker	*Reference*		*Reference*		*Reference*		*Reference*	
Smoker	0.04 (-0.04, 0.11)	0.366	0.03 (-0.06, 0.12)	0.524	2.32 (-4.95, 9.59)	0.526	1.25 (-7.77, 10.28)	0.780
**Patient-Reported Outcomes**								
*Asthma Control Questionnaire (ACQ)*								
*Well controlled*	*Reference*		*Reference*		*Reference*		*Reference*	
Intermediate	-0.09 (-0.18, 0.00)	0.055	-0.06 (-0.17, 0.05)	0.261	0.10 (-8.20, 8.40)	0.981	5.41 (-5.02, 15.84)	0.300
Not well controlled	-0.18 (-0.28, -0.09)	<0.001	-0.15 (-0.26, -0.04)	0.011	-9.83 (-18.47, -1.19)	0.026	-6.68 (-17.77, 4.40)	0.230
**Asthma medication**								
*Type of controller medication*								
Inhaled Corticosteroids (ICs)	*Reference*		*Reference*		*Reference*		*Reference*	
LABA with/out ICs	-0.04 (-0.20, 0.11)	0.583	-0.05 (-0.22, 0.11)	0.496	-3.18 (-17.87, 11.51)	0.667	1.10 (-14.73, 16.93)	0.889
ICs/LABA Fixed-dose combination	-0.02 (-0.13, 0.10)	0.765	0.02 (-0.11, 0.16)	0.738	-1.27 (-11.64, 9.09)	0.807	7.63 (-5.73, 20.98)	0.254
*Adherence (MIS-A 1-week)*								
Complete (100%)	*Reference*		*Reference*		*Reference*		*Reference*	
Intermediate	-0.04 (-0.13, 0.05)	0.402	-0.02 (-0.13, 0.09)	0.708	-0.88 (-9.37, 7.62)	0.838	3.23 (-7.76, 14.21)	0.555
Low (≤50%)	-0.10 (-0.19, -0.01)	0.033	0.04 (-0.06, 0.14)	0.440	-4.93 (-13.47, 3.61)	0.253	6.13 (-3.90, 16.16)	0.223
*Reliever medication use*								
Twice a week or less	*Reference*		*Reference*		*Reference*		*Reference*	
More than twice a week	-0.04 (-0.17, 0.09)	0.552	-0.10 (-0.21, 0.01)	0.074	0.65 (-11.38, 12.68)	0.914	-7.05 (-17.83, 3.73)	0.193
**Severity markers**								
*Asthma-related comorbidities*								
0	*Reference*		*Reference*		*Reference*		*Reference*	
1	0.03 (-0.07, 0.14)	0.542	0.01 (-0.11, 0.13)	0.845	2.39 (-7.27, 12.05)	0.623	-6.57 (-18.28, 5.13)	0.262
2 or more	-0.08 (-0.19, 0.03)	0.153	-0.02 (-0.14, 0.09)	0.677	-2.07 (-12.32, 8.18)	0.688	-3.44 (-15.05, 8.17)	0.552
*Inhaled Corticosteroids daily dose*^*1*^								
*≤ 500 μcg*	*Reference*		*Reference*		*Reference*		*Reference*	
500–1000 μcg	0.08 (-0.02, 0.18)	0.127	-0.08 (-0.18, 0.01)	0.082	12.20 (2.87, 21.52)	0.011	-3.43 (-12.71, 5.86)	0.459
> 1000 μcg	0.01 (-0.10, 0.11)	0.920	-0.08 (-0.21, 0.05)	0.222	3.06 (-6.72, 12.85)	0.534	-7.00 (-19.50, 5.50)	0.264
*Oral Corticosteroids courses*^*2*^								
0 courses	*Reference*		*Reference*		*Reference*		*Reference*	
1 or more courses	0.01 (-0.07, 0.08)	0.839	-0.05 (-0.16, 0.06)	0.353	-2.48 (-9.57, 4.60)	0.486	-2.38 (-13.13, 8.37)	0.656

1 Inhaled corticosteroids prescribed at the time of inclusion (beclomethasone equivalent)

2 Oral corticosteroids courses prescribed during the 12 months before inclusion

## Discussion

This is the first study to examine the impact of asthma on HRQoL considering population reference norms, which allows to estimate asthma burden and to identify high risk groups, incorporating a gender perspective. We found that asthmatic patients consistently reported worse HRQoL than subjects of the same age and gender from the general population, with younger women being the most affected. We identified asthma control as the major contributor to impaired HRQoL in both women and men, while education, medication, and severity markers did not contribute significantly. Translating these differences from reference norms to QALYs, they are interpretable as a mean of 40 fewer days of full health per year experienced by persons with asthma: ranging from 68 in the youngest women (18–24 years) to 27 in the oldest (35–40 years), and from 18 to 31 in men within the same age groups.

Our findings are in agreement with studies based on National Health Surveys, showing that subjects self-reporting asthma have worse HRQoL than those without this condition [14] or the general population [15]. The impact of asthma refers to **how much** patients’ symptoms, functional status and associated diseases matter to them and adversely affect their HRQoL. Beyond statistical significance, there are a number of approaches to interpret the magnitude of differences (‘how much’), such as the Minimum Important Difference (MID) and effect size (difference of means/SD of total sample). The MID is instrument-specific (established in +/-0.07 units for the EQ-5D [35]), while the effect size is not (0.2 SD small, 0.5 SD moderate, and 0.8 SD large [37]). In this study, the negative deviations from reference norms in all the groups evaluated (ranging from -0.075 to -0.181) were equal or higher than the MID, except for men aged 18–24 years, with a deviation of -0.05. In terms of effect size, the magnitude of the difference between women with asthma aged 18–24 years and their counterparts was large (1.1 SD), small in men of this age group (0.29 SD), and moderate in the rest of age/gender groups.

**Fig 3:**
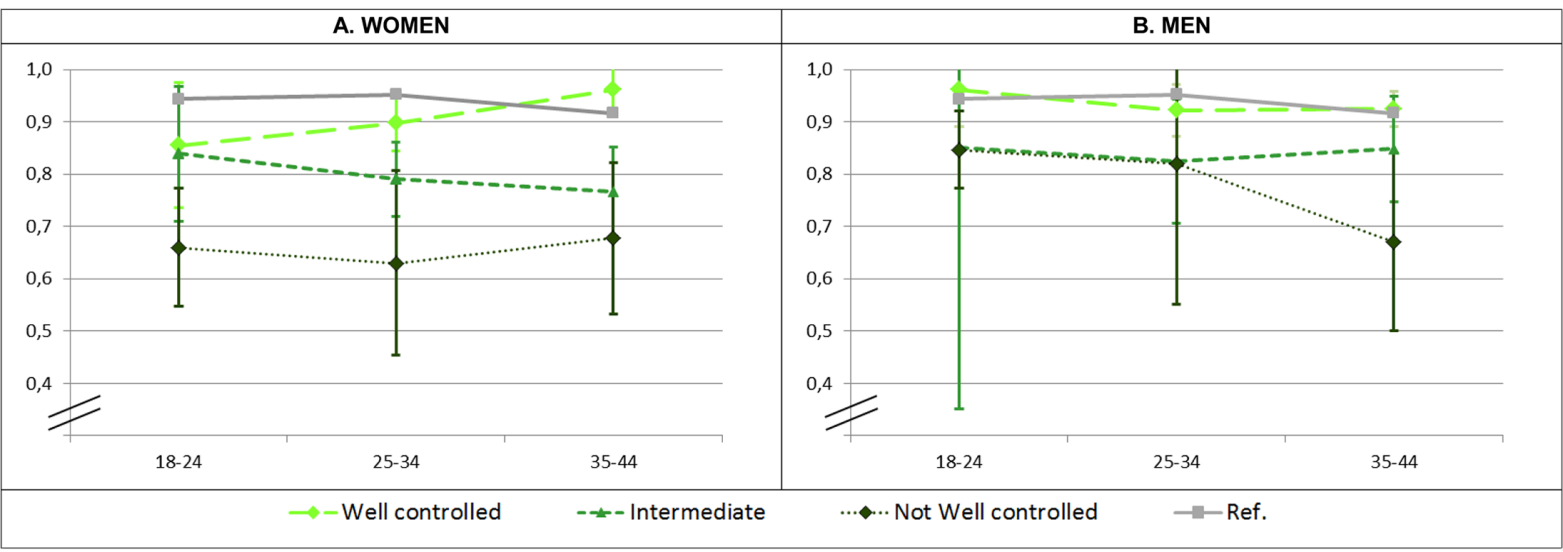
EQ-5D index in patients with asthma, stratified by level of control as measured with ACQ. ACQ = Asthma Control Questionnaire. Well controlled asthma defined as a ACQ score <0.75; intermediate asthma control as ACQ 0.75–1.5; and not well controlled as ACQ score >1.5 [31]. Green dotted lines represent mean and 95% Confidence Interval (95%CI) of EQ-5D index in patients with asthma. Grey continuous line represents the mean in French general population-based reference norms [20].

Our results highlight that asthma control is the most relevant factor to explain impact on HRQoL. Fig 3 shows the distance between our sample and reference norms according to asthma control. These findings are in agreement with the 2008 European National Health and Wellness Survey [15] and a randomly selected cohort with clinical examination [38], in which well-controlled asthma patients presented similar SF-12 scores to the general population.

In our sample subjects with well-controlled asthma also presented a negligible deviation in the EQ-5D index. In contrast, EQ-5D index of patients with uncontrolled asthma was markedly lower than normative values with regression β coefficients of -0.18 in women and -0.15 in men, both far from the MID of +/-0.07 and indicating large impact (effect sizes of 0.88 SD and 1.17 SD, respectively). Translating these regression β coefficients into QALYs, they are interpretable as 66 and 55 fewer days of full health per year in women and men with uncontrolled asthma, respectively, compared with those with controlled asthma.

Previous clinical studies with the traditional EQ-5D reported a very similar mean index to ours: 0.91 vs 0.88 [26] and 0.91 [39] for patients with well-controlled asthma, 0.69 vs 0.61 [26] and 0.73 [39] for those with not well-controlled. Furthermore, a cohort of 8,111 asthmatic patients reported a difference of around 2 points of the Mini Asthma Quality of Life Questionnaire (MiniAQLQ) between those with well-controlled and not well-controlled asthma; this is substantially higher than the +/-0.5 points MID established for the MiniAQLQ [25]. Significant associations between severity markers and HRQoL disappeared after introducing asthma control in the multivariate models. This supports that control could be a mediator factor between severity and HRQoL. These consistent results suggest that the impact of asthma on HRQoL could be alleviated by achieving a good asthma control, reinforcing the relevance of its close follow-up.

Women in the general population have consistently presented worse HRQoL than men despite [40,41], paradoxically, having a higher life expectancy. Studies in clinical samples of asthma patients also reported that HRQoL impairment is greater among women than men [2–4]. Nevertheless, this is the first study confirming that the impact of asthma on HRQoL is higher in women during early adulthood (18–24 years), as deviations from general population-based reference norms indicated a large impact for women (1.1 SD) and small for men (0.29 SD). In this sense, it is important to highlight that, compared to men, this group of very young women had more severe asthma (mean inhaled corticosteroids daily dose 1302.9 vs 835.7 μcg, and number of oral corticosteroids courses 0.61 vs 0.26, p = 0.179 and 0.096 respectively), worse asthma control (mean ACQ score 1.4 vs 1.1, p = 0.449) and lower medication adherence (66.5% vs 56.1%, p = 0.349), but differences were not statistically significant due to the small sample size (36 women and 19 men). Impact of asthma in the youngest women (18–24 years) merits further research to identify explanatory factors (e.g. hormonal, physical activity) underlying this large asthma HRQoL impact at this first stage of women’s adult life.

Our study showed that the impact of asthma on patients’ HRQoL is moderate in most age-gender groups studied. This impact is greater than other chronic conditions previously evaluated with this approach, such as type 2 diabetes mellitus [13], epilepsy [12], and thalassemia [11], but lower than that of rheumathoid arthritis in the physical component of health [10]. The impact of type 2 diabetes mellitus was small, deviation of EQ-5D index from general population only reached the MID of +/-0.07 units in patients aged 55–64 years (-0.085) [13], while the youngest presented lower deviations. Similarly, the 36-item Short-Form Health Survey (SF-36) indicated that the impact of epilepsy on HRQoL was small in role physical and emotional (effect sizes of 0.29 and 0.42) [12], and that of thalassemia was small on the physical and mental health components [11] (effect sizes of 0.32 and 0.20). Rheumatoid arthritis presented a large impact on physical health and a moderate one on mental health [10], as measured with SF-36 component summaries (effect sizes of 1.8 and 0.6).

It is important to remark that the real impact of asthma on HRQoL could be even higher than described here. Since general population includes a proportion of patients with asthma (as well as other diseases), the differences between our asthma sample and EQ-5D reference norms would have been greater than observed if we strictly compared with subjects without asthma. The most prevalent chronic conditions reported by French individuals aged 15–39 years in the European Health Interview Survey—‘Enquête Santé et Protection Sociale’ (EHIS-ESPS) 2014 [42] were: low back pain (19.8%), allergies (15.9%), cervical pain (9.0%), asthma (8.4%), diabetes (4.2%) and depression (4.1%). As expected, the prevalence of asthma-related chronic conditions was higher in our sample (allergic rhinitis 48.4% and depression 15.3%), but for those non-related to asthma such as musculo-skeletal conditions and diabetes prevalence was not expected to differ from EHIS-ESPS 2014. Although information on non-asthma-related comorbidity was not collected in ASTRO-LAB project, the young age of participants in our study (18–40 years) makes less likely confounding the impact of asthma on HRQoL with other comorbid conditions. For example, prevalence of arthritis in the EHIS-ESPS 2014 [42] was 1.7% in the age group of 15–39 years old, 20.0% in the group of 40–64 years, and 49.5% in the group of 65 or more years.

Some potential limitations of the current study need to be considered. First, our findings cannot establish causality between asthma control and HRQoL because of its cross-sectional nature; therefore, we cannot rule out reverse causality. In this sense, when we use the term ‘asthma impact’ we are referring to the impairment associated with asthma, we are not suggesting causality. Second, even though we adjusted for severity with three markers, two of them based on drug prescription and one on asthma-related comorbidity, there still might be a residual confounding. Third, although the online survey participation rate was low (49%), the only significant difference between respondents and non-respondents was found in the asthma control questionnaire: non-respondents reported less symptom control; therefore, our results might underestimate the impact of asthma on HRQoL (see Table 4). Finally, because our study only included 18–40 year-old adults receiving daily treatment with inhalers, the generalisability of our results to those older than 40 years and/or with intermittent treatment is uncertain.

**Table 4:** Characteristics in respondents and non-respondents to the EQ-5D-5L.

	EQ-5D respondents (n = 222)	EQ-5D non-respondents (n = 265)	p
Gender			
Women	136 (61.3%)	150 (56.6%)	
Men	86 (38.7%)	115 (43.4%)	0.298
Age. mean (SD)	30.3 (6.7)	29.5 (6.6)	0.179
18–24 years	55 (24.8%)	78 (29.4%)	0.387
25–35 years	97 (43.7%)	116 (43.8%)	
35 or more years	70 (31.5%)	71 (26.8%)	
**Patient-reported outcomes**			
Asthma control Questionnaire (ACQ), mean (SD)	1.1 (1.0)	1.3 (1.0)	0.048
Well-controlled (< 0.75)	67 (37.9%)	83 (35.6%)	0.010
Intermediate (0.75–1.5)	61 (34.5%)	55 (23.6%)	
Not well-controlled (> 1.5)	49 (27.7%)	95 (40.8%)	
*Missing*	45	32	
**Asthma medication**			
Type of controller medication			
Inhaled Corticosteroids (ICs)	39 (17.6%)	43 (16.2%)	0.666
Long-acting beta-agonist (LABA) with/out ICs	30 (13.5%)	30 (11.3%)	
ICs/LABA fixed combination	153 (68.9%)	192 (72.5%)	
Adherence (MIS-A 1-week)			
Low (≤50%)	57 (30.3%)	52 (28.1%)	0.192
Intermediate	50 (26.6%)	65 (35.1%)	
Complete (100%)	81 (43.1%)	68 (36.8%)	
Missing	34	80	
Reliever medication use			
Twice a week or less	189 (89.6%)	209 (89.7%)	0.965
More than twice a week	22 (10.4%)	24 (10.3%)	
Missing	11	32	
**Severity Markers**			
Asthma-related comorbidities			
0	66 (41.5%)	80 (39.2%)	0.511
1	62 (39.0%)	91 (44.6%)	
2 or more	31 (19.5%)	33 (16.2%)	
Inhaled Corticosteroids daily dose^1^, mean (SD)	929.8 (866.2)	942.0 (823.9)	0.883
≤ 500 μg	89 (44.1%)	88 (41.5%)	0.674
500–1000 μg	65 (32.2%)	77 (36.3%)	
> 1000 μg	48 (23.8%)	47 (22.2%)	
Oral Corticosteroids courses^2^, mean (SD)	0.4 (0.8)	0.5 (1.0)	0.905
0	152 (70.4%)	186 (72.9%)	0.537
1 or more	64 (29.6%)	69 (27.1%)	

1 Inhaled corticosteroids prescribed at the time of inclusion (beclomethasone equivalent)

2 Oral corticosteroids courses prescribed during the 12 months before inclusion

## Conclusions

Findings confirm our hypothesis that the worse HRQoL in women with asthma compared with men [2–4] seems not to imply real gender inequalities in asthma impact, except for the youngest age group. Our results support considering very young women (18–24 years old) a high-risk group. Therefore, the large HRQoL impact of asthma in this group calls for closer monitoring of symptoms control, asthma self-management programs and adequate medical therapy. In general, persistent asthma has a moderately negative HRQoL impact on patients of both genders at an adult age (25–40 years old). Our study identifies asthma control as the main factor associated to HRQoL, suggesting that its improvement could alleviate the large HRQoL impairment found in women and men with uncontrolled asthma. Effective support options need to be explored for groups at high risk of suffering a large negative asthma impact on HRQoL.

## Supporting information

S1 Dataset Study anonymized dataset. (XLSX)
